# Pre-sleep Protein Ingestion Increases Mitochondrial Protein Synthesis Rates During Overnight Recovery from Endurance Exercise: A Randomized Controlled Trial

**DOI:** 10.1007/s40279-023-01822-3

**Published:** 2023-03-01

**Authors:** Jorn Trommelen, Glenn A. A. van Lieshout, Pardeep Pabla, Jean Nyakayiru, Floris K. Hendriks, Joan M. Senden, Joy P. B. Goessens, Janneau M. X. van Kranenburg, Annemie P. Gijsen, Lex B. Verdijk, Lisette C. P. G. M. de Groot, Luc J. C. van Loon

**Affiliations:** 1 Department of Human Biology, NUTRIM School of Nutrition and Translational Research in Metabolism, Maastricht University Medical Centre+, P.O. Box 616, 6200 MD Maastricht, The Netherlands; 2 FrieslandCampina, 3818 LE Amersfoort, The Netherlands; 3 MRC/Versus Arthritis Centre for Musculoskeletal Ageing Research, School of Life Sciences, University of Nottingham, Nottingham, UK; 4 Division of Human Nutrition, Wageningen University, Wageningen, The Netherlands

## Abstract

**Background:**

Casein protein ingestion prior to sleep has been shown to increase myofibrillar protein synthesis rates during overnight sleep. It remains to be assessed whether pre-sleep protein ingestion can also increase mitochondrial protein synthesis rates. Though it has been suggested that casein protein may be preferred as a pre-sleep protein source, no study has compared the impact of pre-sleep whey versus casein ingestion on overnight muscle protein synthesis rates.

**Objective:**

We aimed to assess the impact of casein and whey protein ingestion prior to sleep on mitochondrial and myofibrillar protein synthesis rates during overnight recovery from a bout of endurance-type exercise.

**Methods:**

Thirty-six healthy young men performed a single bout of endurance-type exercise in the evening (19:45 h). Thirty minutes prior to sleep (23:30 h), participants ingested 45 g of casein protein, 45 g of whey protein, or a non-caloric placebo. Continuous intravenous l-[ring-^13^C_6_]-phenylalanine infusions were applied, with blood and muscle tissue samples being collected to assess overnight mitochondrial and myofibrillar protein synthesis rates.

**Results:**

Pooled protein ingestion resulted in greater mitochondrial (0.087 ± 0.020 vs 0.067 ± 0.016%·h^−1^, *p* = 0.005) and myofibrillar (0.060 ± 0.014 vs 0.047 ± 0.011%·h^−1^, *p* = 0.012) protein synthesis rates when compared with placebo. Casein and whey protein ingestion did not differ in their capacity to stimulate mitochondrial (0.082 ± 0.019 vs 0.092 ± 0.020%·h^−1^, *p* = 0.690) and myofibrillar (0.056 ± 0.009 vs 0.064 ± 0.018%·h^−1^, *p* = 0.440) protein synthesis rates.

**Conclusions:**

Protein ingestion prior to sleep increases both mitochondrial and myofibrillar protein synthesis rates during overnight recovery from exercise. The overnight muscle protein synthetic response to whey and casein protein does not differ.

**Clinical Trial Registration:**

NTR7251.

**Supplementary Information:**

The online version contains supplementary material available at 10.1007/s40279-023-01822-3.

## Key Points

**Table:** 

Protein ingestion prior to sleep increases myofibrillar and mitochondrial protein synthesis rates during overnight recovery from endurance-type exercise.
The overnight muscle protein synthetic response to whey and casein ingestion does not differ.
Pre-sleep protein ingestion facilitates the skeletal muscle adaptive response to exercise.
This is the first study to show that protein ingestion during recovery from exercise increases mitochondrial protein synthesis rates.

## Introduction

We have previously demonstrated that protein ingested prior to sleep is effectively digested and absorbed [1–3], stimulates overnight muscle protein synthesis [1, 3], and allows the overnight whole-body protein net balance to become positive [1–3]. In addition, protein supplementation prior to sleep increases muscle mass and strength gains during prolonged resistance-type exercise training [4]. Therefore, pre-sleep protein ingestion is now widely recommended as a strategy to improve overnight recovery and facilitate the skeletal muscle adaptive response to exercise training [5–7].

It is often suggested that micellar casein is the preferred type of protein prior to sleep to stimulate overnight anabolism. Micellar casein is a slowly digestible protein that clots in the stomach, resulting in a moderate but more sustained postprandial release of protein-derived amino acids [8, 9]. In support, pre-sleep casein ingestion has been shown to increase plasma amino acid levels throughout the entire overnight period [1–3]. While pre-sleep casein ingestion has been shown to stimulate overnight anabolism [1, 3], its efficacy to increase overnight muscle protein synthesis rates has not been compared to other protein sources. Whey protein is generally considered the highest quality protein source [10]. Whey is a more rapidly digestible protein and its ingestion results in a rapid but more transient postprandial increase in plasma amino acid levels [8, 9]. Furthermore, whey protein has a higher essential amino acid content and provides more leucine when compared with an isonitrogenous amount of casein protein [11]. Various studies have compared the muscle protein synthetic response to whey and casein protein ingestion [12–18]. While two studies demonstrated greater postprandial muscle protein synthesis rates following the ingestion of whey compared with casein protein [14, 18], most studies have failed to detect significant differences [12, 13, 15–17]. To date, no studies have compared whey and casein protein in their capacity to stimulate overnight muscle protein synthesis.

It has been well established that protein ingestion during recovery from exercise increases myofibrillar protein synthesis rates [19–22]. It is far less evident whether protein ingestion during recovery from exercise can also increase mitochondrial protein synthesis rates. Previous studies have not been able to detect a significant impact of post-exercise protein ingestion on mitochondrial protein synthesis rates [12, 20–24]. However, there may be a latency in the exercise-induced increase in mitochondrial when compared with myofibrillar protein synthesis rates [21, 25–27]. Whereas myofibrillar protein synthesis rates are typically highest during acute post-exercise recovery (0–6 h) [28, 29], mitochondrial protein synthesis rates appear to peak at ~ 24 h of post-exercise recovery [25, 27, 30]. Therefore, it could be speculated that post-exercise protein ingestion may prove to be more effective at stimulating mitochondrial protein synthesis rates when assessed over a more prolonged recovery period [31].

The present study assesses the impact of protein ingestion prior to sleep on overnight myofibrillar and mitochondrial protein synthesis rates following a single bout of endurance-type exercise in healthy young adults. Here, we compare the efficacy of both whey and casein protein ingestion to increase muscle protein synthesis rates during overnight sleep.

## Methods

### Participants

A total of 36 healthy young men were selected to participate in this study (Table 1). Participants were recruited through social media and dedicated bulletin boards within Maastricht University. Inclusion criteria were: male, aged between 18 and 35 years, healthy, and body mass index between 18.5 and 30 kg·m^−2^. Exclusion criteria were: smoking, sports/exercise < 1 or > 3 sessions per week, lactose intolerant, a history of neuromuscular problems, use of anticoagulation medication, recent (< 1 year) participation in ^13^C_6_-phenylalanine tracer studies, and individuals taking any medication known to affect protein metabolism. Participants were randomly assigned to ingest 0 g of protein (PLA, *n* = 12), 45 g of casein protein (CAS, *n* = 12), or 45 g of whey protein (WHEY, *n* = 12) prior to sleep. The experiments were performed in a randomized double-blinded manner. All participants were fully informed of the nature and possible risks of the experimental procedures before their written informed consent was obtained. The project was registered at Nederlands Trial Register as NTR7251 and was approved by the Medical Ethical Committee of Maastricht University Medical Centre, Maastricht, the Netherlands, and conformed to standards for the use of human participants in research as outlined in the most recent version of the Helsinki Declaration. The study was independently monitored by the Clinical Trial Center Maastricht.

**Table 1:** Subjects’ characteristics

	PLA (*n* = 12)	CAS (*n* = 12)	WHEY (*n* = 12)	ALL (*n* = 36)
Age (y)	26 ± 5	25 ± 4	24 ± 3	25 ± 4
Body mass (kg)	76.4 ± 7.8	76.2 ± 5.5	78 ± 11	76.8 ± 8.2
BMI (kg m^−2^)	22.7 ± 1.3	23.6 ± 1.8	23.6 ± 2.7	23.3 ± 2
LBM (kg)	62 ± 5.8	60.1 ± 4.8	61.1 ± 8	61.1 ± 6.2
Fat mass (kg)	15.4 ± 4.3	16.5 ± 3.2	17.4 ± 5.2	16.4 ± 4.3
Fat (%)	20 ± 5	22 ± 4	22 ± 4	21 ± 4
VO_2peak_ (ml kg^−1^ min^−1^)	51 ± 9	47 ± 8	47 ± 7	48 ± 8
W_max_ (W kg^−1^)	4.4 ± 0.9	4 ± 0.8	3.8 ± 0.8	4 ± 0.8
Energy intake (MJ d^−1^)	10.5 ± 1	10.9 ± 1.2	10.8 ± 2.1	10.7 ± 1.5
Protein intake (g·d-1)	1.3 ± 0.3	1.4 ± 0.3	1.3 ± 0.3	1.3 ± 0.3
Protein (% of energy)	16 ± 4	16 ± 4	16 ± 5	16 ± 4
Carbohydrate (% of energy)	48 ± 3	46 ± 7	45 ± 7	46 ± 6
Fat intake (% of energy)	34 ± 3	35 ± 5	36 ± 6	35 ± 5

Values are means+SD

*PLA* placebo treatment,* CAS* micellar casein protein treatment,* WHEY* whey protein treatment

### Pretesting

Bodyweight and body composition were determined by dual-energy x-ray absorptiometry (Discovery A; Hologic, Bedford, MA, USA). Participant’s maximal workload capacity and peak oxygen consumption were determined while performing a stepwise exercise test to exhaustion on an electronically braked cycle ergometer (Lode Excalibur, Groningen, The Netherlands), using an online gas-collection system (Omnical, Maastricht University, Maastricht, The Netherlands). After a 5-min warm up at 100 W, workload was set at 150 W and increased 50 W every 2.5 min until exhaustion. Peak oxygen consumption was defined as the median of the highest consecutive values over 30 s. Maximal workload capacity was calculated as the workload in the last completed stage and workload relative to the time spent in the last incomplete stage: (time in seconds)/150 × 50 (W). The pretesting and experimental trials were separated by at least 7 days.

### Diet and Physical Activity

All participants were instructed to refrain from exhaustive physical labor and exercise and to keep their diet as constant as possible 2 days before the experimental day. Food intake and physical activity questionnaires were collected for 2 days before the experiment. All participants received a standardized diet throughout the experimental day [0.16 MJ·kg^−1^, providing 64 energy percentage (En%) carbohydrate, 12 En% protein, and 24 En% fat]. The energy content of the standardized diet was based on individual energy requirements based on the Harris–Benedict equation and adjusted using a physical activity factor of 1.6 to ensure ample energy intake. During the experimental day, all participants ingested 1.2 ± 0.1 g of protein·kg body mass^−1^ via the standardized diet. Participants receiving the casein or whey protein treatment ingested an additional 45 g (0.6 g·kg^−1^) of protein before sleep (23:30 h). Prior to intake of the test drink and in the morning after the intervention, hunger and sleep ratings were assessed using the visual analog scale [32]. Subjects indicated their feeling of hunger and sleep on 10-cm lines, with the left side (0 cm) representing “absolutely no hunger” and “absolutely no sleep” and the right side (10 cm) representing “extremely hungry” and “extremely sleepy”. In the morning, participants were invited to an ad libitum breakfast, and food intake at breakfast was recorded for all participants.

### Test Beverages

Whey protein concentrate (Nutri Whey™ 800F) and micellar casein protein isolate (Refit MCI^MF^ 88) were obtained from FrieslandCampina Ingredients. The proteins had minimal glycation (blocked lysine < 5%) and high solubility (> 95%) [33, 34]. The WHEY and CAS drinks contained 3% free l-[ring-^13^C_6_]-phenylalanine to minimize dilution of the steady-state plasma l-[ring-^13^C_6_]-phenylalanine precursor pool implemented by the constant infusion [35]. Beverages contained 450 mL of water and 1.5 mL of vanilla extract (Dr. Oetker, Amersfoort, The Netherlands).

### Experimental Protocol

**Fig. 1:**
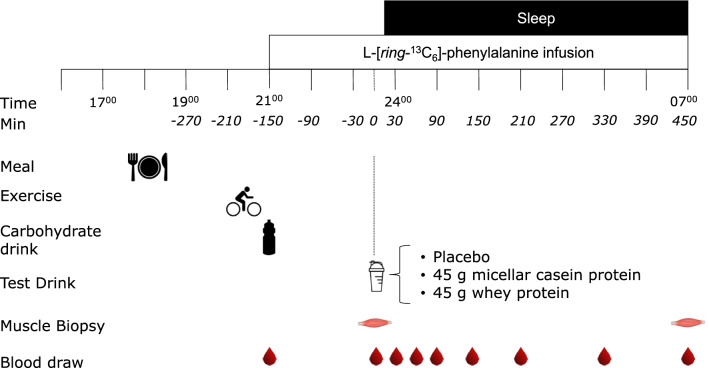
Experimental protocol. A carbohydrate drink was consumed immediately following a bout of endurance-type exercise at 20:45 h. Participants were randomly assigned to ingest placebo, 45 g of casein protein, or 45 g of whey protein at 23:30 h

At 17:00 h, participants reported to the laboratory. At 17:30 h (*t* =  − 360 min), the participants consumed a standardized dinner (0.04 MJ·kg^−1^, providing 69 En% carbohydrate, 15 En% protein, and 16 En% fat; Sligro, Maastricht, The Netherlands). A single bout of endurance-type exercise was performed between 19:45 and 20:45 h. A 330-mL drink providing 49 g of carbohydrate was ingested immediately after exercise and ingested within 2 min (AA drink High Energy, United Soft Drinks, Utrecht, The Netherlands). Catheters were inserted into an antecubital vein of each arm. Subsequently, a background blood sample was taken before the initiation of the tracer infusion protocol, which was started at 21:00 h (*t* =  − 150 min). Plasma and intracellular phenylalanine pools were primed with a single intravenous dose (priming dose) of l-[ring-^13^C_6_]-phenylalanine (2.25 μmol·kg^−1^). Once primed, the continuous stable isotope infusion was initiated (infusion rate: 0.05 μmol·kg^−1^·min^−1^ l-[ring-^13^C_6_]-phenylalanine (Cambridge Isotopes Laboratories, Andover, MA, USA). Participants rested in a supine position for 2.5 h until 23:30 h, after which the first muscle biopsy was taken. Subsequently, participants ingested the beverage (*t* = 0 min) PLA, CAS, or WHEY within 5 min. Following drink ingestion, participants prepared to go to sleep (sleep time: ~ 00:00 h). During the night, blood samples (10 mL) were taken without waking up the participants at *t* = 30, 60, 90, 150, 210, 330, and 450 min relative to the intake of the protein drink. Participants were woken at ~ 07:00 h after which the second muscle biopsy was from the contralateral leg (*t* = 450 min) (Fig. 1).

Blood samples were collected in EDTA-containing tubes and centrifuged at 1000* g* for 10 min at 4 °C. Aliquots of plasma were frozen in liquid nitrogen and stored at − 80 °C. Muscle biopsies were obtained from the middle region of the musculus vastus lateralis, 15 cm above the patella and approximately 4 cm below entry through the fascia, using the percutaneous needle biopsy technique [36]. Muscle samples were dissected carefully and freed from any visible non-muscle material. The muscle samples were immediately frozen in liquid nitrogen and stored at − 80 °C until further analysis.

### Exercise Protocol

Participants performed 60 min of continuous endurance exercise on a cycle ergometer at a fixed 60% of their previously determined maximal workload capacity. Participants were allowed ad libitum access to water during cycling. Visual feedback for pedal frequency (revolutions/min) and elapsed time were provided to participants.

### Plasma and Muscle Analyses

Plasma glucose and insulin levels were measured using commercially available kits (GLUC3; Roche, Basel, Switzerland, Ref: 05,168,791,190 and Immunologic; Roche, Ref: 12,017,547,122, respectively). Plasma amino acid levels were quantified using ultra performance liquid chromatography-mass spectrometry as described previously [33]. Plasma phenylalanine levels and l-[ring-^13^C_6_]-phenylalanine enrichments were determined by GC–MS (Agilent 7890A GC/5975C; MSD) as described previously [37]. Myofibrillar and mitochondrial protein-bound phenylalanine enrichments were determined as described previously [12]. In separate pieces of muscle (*n* = 3), myofibrillar and mitochondrial protein (maximal) citrate synthase activity was measured as described before [38]. Enzyme activity is expressed as micromoles of product (citrate) generated per gram of protein per minute during the assay (µmol·g protein^−1^·min^−1^). Protein content in the muscle fractions was assessed by a bicinchoninic acid assay on both the supernatant and pellet. Western blots were performed on the myofibrillar and mitochondrial protein fractions to determine their purity. A portion of each muscle sample frozen for biochemical analyses was homogenized in seven volumes of Tris buffer (20 mm of Tris–HCL, 5 mm of EDTA, 10 mm of Na-pyrophosphate, 100 mm of NaF, 2 mm of Na_3_VO_4_, 1% Nonident P-40; pH 7.4) supplemented with protease and phosphatase inhibitors: 10 µg mL^–1^ of aprotinin, 10 µg mL^–1^ of leupeptin, 3 mm of benzamidine, and 1 mm of phenylmethylsulphonyl fluoride. After homogenization, each muscle extract was centrifuged for 10 min at 10,000*g* (4 °C) and a sample buffer was added to the supernatant to final concentrations of 60 mm of Tris, 10% glycerol, 20 mg mL^–1^ of SDS, 0.1 mm of dithiothreitol, and 20 µg mL^–1^ of bromophenol blue. The supernatant was then heated for 5 min at 100 °C and immediately placed on ice. Immediately before analyses, the muscle extraction sample was warmed to 50 °C and centrifuged for 1 min at 1000*g* (room temperature). The total amount of sample loaded on the gel was based on the protein content. After a Bradford assay, an equal amount of protein was loaded on the gel. Samples were run on a Criterion Stain-Free TGX 4–15% gel (Order No. 567–8083; Bio-Rad, Hercules, CA, USA) ± 90 min at 150 V (constant voltage) and transferred onto a Trans-blot Turbo 0.2 µm nitrocellulose membrane (Order No. 170-4159; Bio-Rad) for 10 min at 2.5 A and 25 V. Specific proteins were detected by overnight incubation at 4 °C on a shaker with specific antibodies in 50% in PBS Odyssey blocking buffer (Part No. 927-40000; Li-Cor Biosciences, Lincoln, NE, USA) after blocking for 60 min at room temperature in 50% in PBS Odyssey blocking buffer. Primary antibodies myosin IIa #49349 and citrate synthase #14309 were purchased from Cell Signaling Technology, Danvers, MA, USA. Following incubation, membranes were washed three times for 10 min in 0.1% PBS-Tween 20 and once for 10 min in PBS. Next, samples were incubated on a shaker (1 h at room temperature) with an infrared secondary antibody donkey anti-rabbit IRDYE 800 (dilution 1:10,000; Cat. No. 611-732-127; Rockland Immunochemicals, Pottstown, PA, USA) dissolved in 50% PBS Odyssey blocking buffer. After a final wash step (3 × 10 min) in 0.1% Tween 20-PBS and once for 10 min in PBS, protein quantification was performed by scanning on an Odyssey Infrared Imaging System (Li-Cor Biosciences). The picture of the stain-free gel was used to standardize for the amount of protein loaded.

### Calculations

The fractional synthetic rate (FSR) of myofibrillar and mitochondrial protein was calculated by dividing the increment in protein fraction enrichment by the precursor amino acid enrichment.

$$\mathrm{FSR }\left(\%{\cdot h}^{-1}\right)=\left(\frac{{E}_{\mathrm{m}2}- {E}_{\mathrm{m}1}}{{E}_{\mathrm{precursor}} \cdot t}\right) \cdot 100\%$$

*E*_m2_* − E*_M1_ represent the increment in myofibrillar or mitochondrial protein-bound l-[ring-^13^C_6_]-phenylalanine enrichments in mole percent excess between the two muscle samples. *E*_precursor_ is the weighted mean plasma l-[ring-^13^C_6_]-phenylalanine enrichment in mole percent excess during the tracer incorporation period, and *t* is the tracer incorporation time in hours.

### Statistics

All data in text are expressed as mean ± standard deviation. Time-dependent variables (i.e., plasma glucose, insulin, amino acid levels, and tracer enrichments) were analyzed by a two-factor repeated-measures analysis of variance (ANOVA) with time as a within-subject factor and treatment group as a between-subject factor. Non-time-dependent variables (i.e., overnight myofibrillar and mitochondrial protein synthesis rates) were compared between treatments using a one-factor ANOVA. A power calculation was performed with differences in overnight myofibrillar protein FSR as the primary outcome measure with the use of a standard deviation of 0.007%·h^−1^ in all treatments, and a difference in FSR of 0.011%·h^−1^ between WHEY and CAS versus PLA (or ~ 33% when expressed as a relative difference between treatments) [1]. With a power of 80% and a significance level of 0.017 (0.05/3 to account for Bonferroni correction), the minimal number of participants to be included was calculated as *n* = 10 per group. To account for potential dropout, we recruited 12 participants per group. Bonferroni post hoc analysis was performed whenever a significant F ratio was found to isolate specific differences. Statistical analyses were performed with a software package (IBM SPSS Statistics for Windows, Version 21.0; IBM Corp., Armonk, NY, USA). Means were considered to be significantly different for *p* values < 0.05.

## Results

### Plasma Glucose and Insulin Levels

**Fig. 2:**
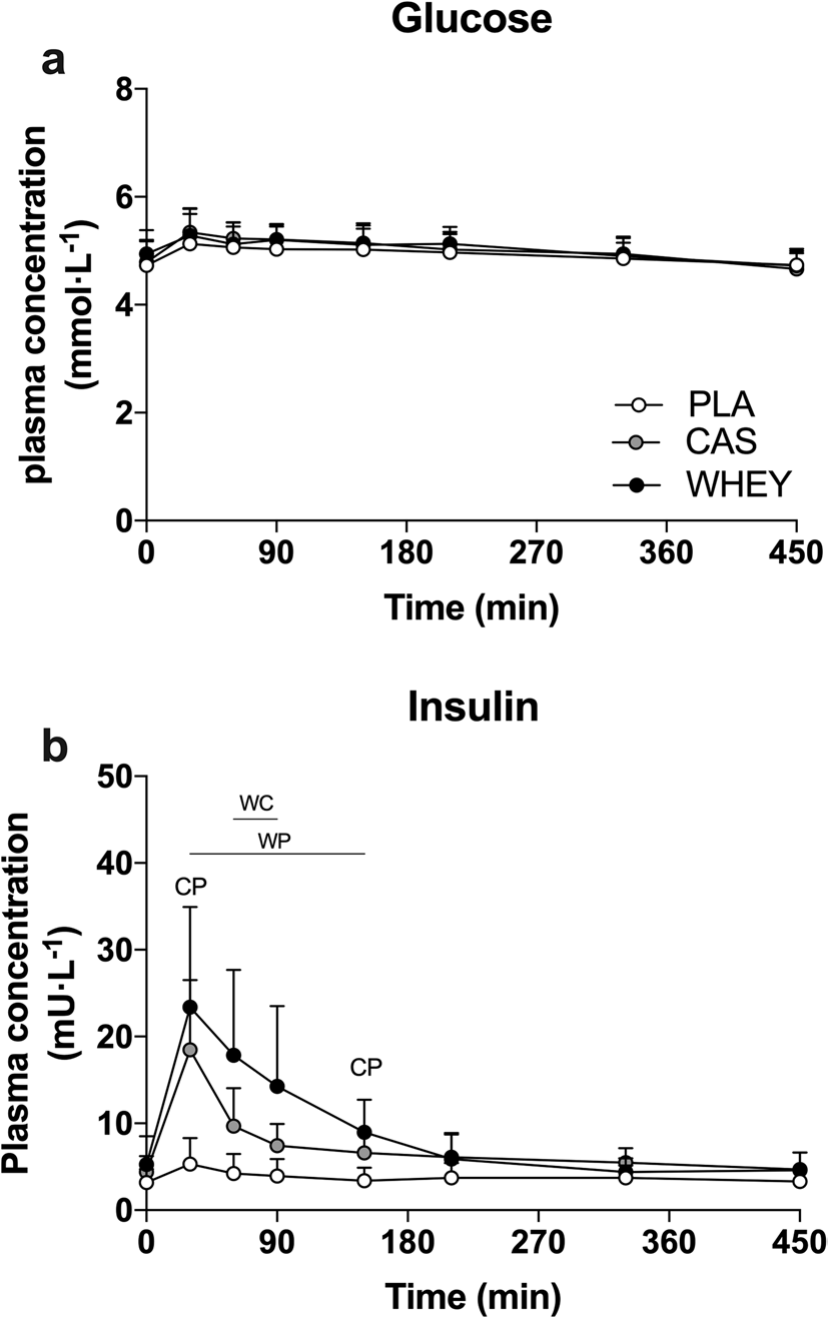
Overnight plasma glucose (**a**) and insulin (**b**) levels following pre-sleep placebo (PLA), casein protein (CAS), or whey protein (WHEY) ingestion. Data are analyzed by two-factor repeated-measures analysis of variance with time as the within-subject factor and treatments as the between-subject factor. The data are expressed as mean ± standard deviation. Time × treatment: panel **a**: *p* = 0.298, panel **b**: *p* < 0.001. ^CP^CAS significantly higher than PLA. ^WP^WHEY significantly higher than PLA. ^WC^WHEY significantly higher than CAS, *p* < 0.05

Plasma glucose and insulin levels are shown in Fig. 2. Plasma glucose levels showed a transient increase at *t* = 30 min (time effect: *p* < 0.001), but did not differ between treatments throughout the overnight period (time*treatment interaction: *p* = 0.298). Plasma insulin levels increased following protein ingestion (time × treatment interaction: *p* < 0.001), with a subsequent analysis showing a more prolonged rise following whey ingestion when compared with casein protein ingestion (*t* = 60–90; ANOVA, *p* < 0.05).

### Plasma Amino Acid Levels

**Fig. 3:**
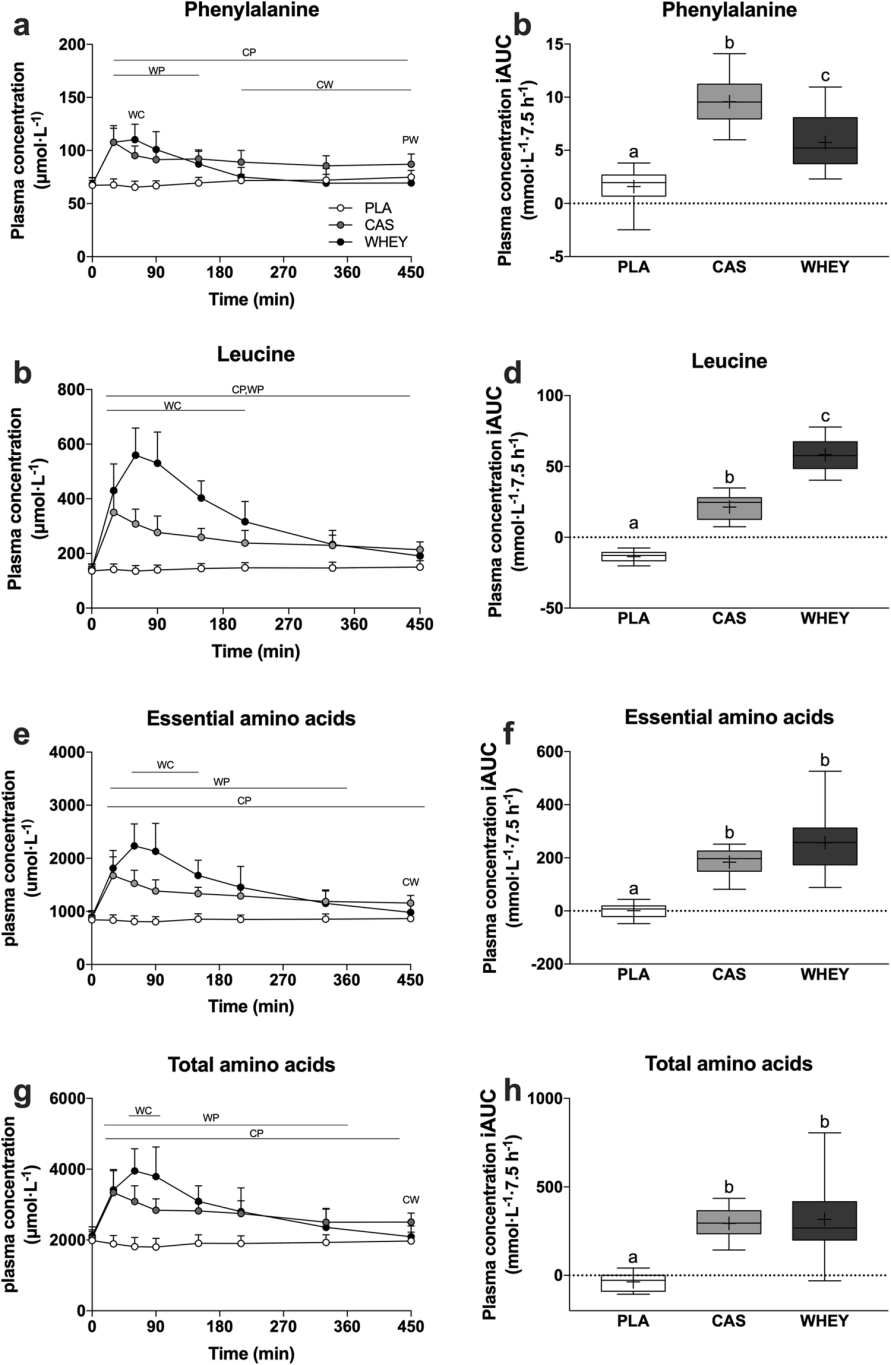
Overnight plasma levels and incremental area under the curve (iAUC) of phenylalanine (**a**, **b**, leucine (**c**, **d**), essential amino acid (**e**, **f**), and total amino acid levels (**g**, **h**) following pre-sleep placebo (PLA), casein protein (CAS), or whey protein (WHEY) ingestion. Timeline data are analyzed by two-factor repeated-measures analysis of variance with time as the within-subject factor and treatments as the between-subject factor. Timeline data are expressed as mean ± standard deviation. Incremental area under the curve data are analyzed with a one-way analysis of variance. Incremental area under the curve data are expressed as box-and-whisker plots with the median (line), mean (cross), interquartile range (box), and minimum and maximum values (tails). Time × treatment: panels **a**, **c**, **e**, **g**: *p* < 0.001. Analysis of variance: panels **b**, **d**, **f**,** h**: *p* < 0.001. ^CP^: CAS significantly higher than PLA. ^WP^WHEY significantly higher than PLA. ^WC^WHEY significantly higher than CAS. iAUC data: treatments without a common letter differ, *p* < 0.05

Plasma amino acid levels and incremental areas under the curves are shown in Fig. 3. Total plasma amino acid levels increased following protein ingestion (time × treatment: *p* < 0.001). Total plasma amino acid levels were higher 60–90 min following the ingestion of whey protein when compared with after casein protein ingestion (*p* < 0.001). In contrast, total plasma amino acid levels upon waking (*t* = 450 min) were significantly higher following casein protein ingestion when compared with whey protein ingestion (*p* = 0.002). Plasma amino acid levels for branched-chain amino acids, non-essential amino acids, and individual amino acids are displayed in Fig. 1 of the Electronic Supplementary Material.

### Plasma **l**-[ring-^13^C_6_] Enrichments

**Fig. 4:**
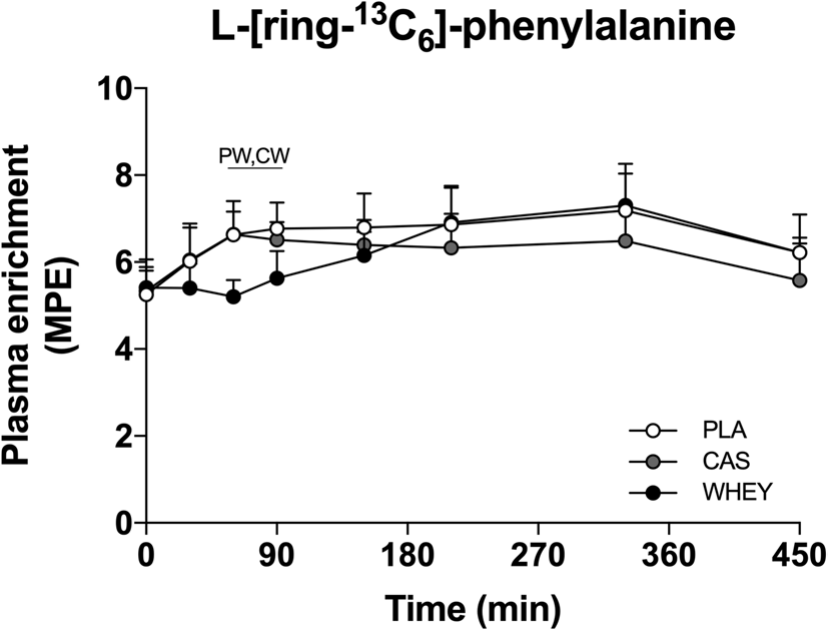
Overnight plasma l-[ring-^13^C_6_]-phenylalanine enrichments following pre-sleep placebo (PLA), casein protein (CAS), or whey protein (WHEY) ingestion. Data are analyzed by two-factor repeated-measures analysis of variance with time as the within-subject factor and treatments as the between-subject factor. The data are expressed as mean ± standard deviation. Time × treatment: *p* < 0.001. ^PW^PLA significantly higher than WHEY. ^CW^CAS significantly higher than WHEY, *p* < 0.05. *MPE* mole percent excess

Plasma l-[ring-^13^C_6_]-phenylalanine enrichments are shown in Fig. 4. Plasma l-[ring-^13^C_6_]-phenylalanine enrichments did not differ between treatments before drink ingestion (*t* = 0 min, *p* = 0.819). Plasma l-[ring-^13^C_6_]-phenylalanine enrichments increased slightly throughout the night (time effect: *p* < 0.001), and this increase was delayed in the WHEY protein treatment when compared with PLA and CAS (time × treatment interaction: *p* < 0.001).

### Muscle Protein Synthesis Rates

**Fig. 5:**
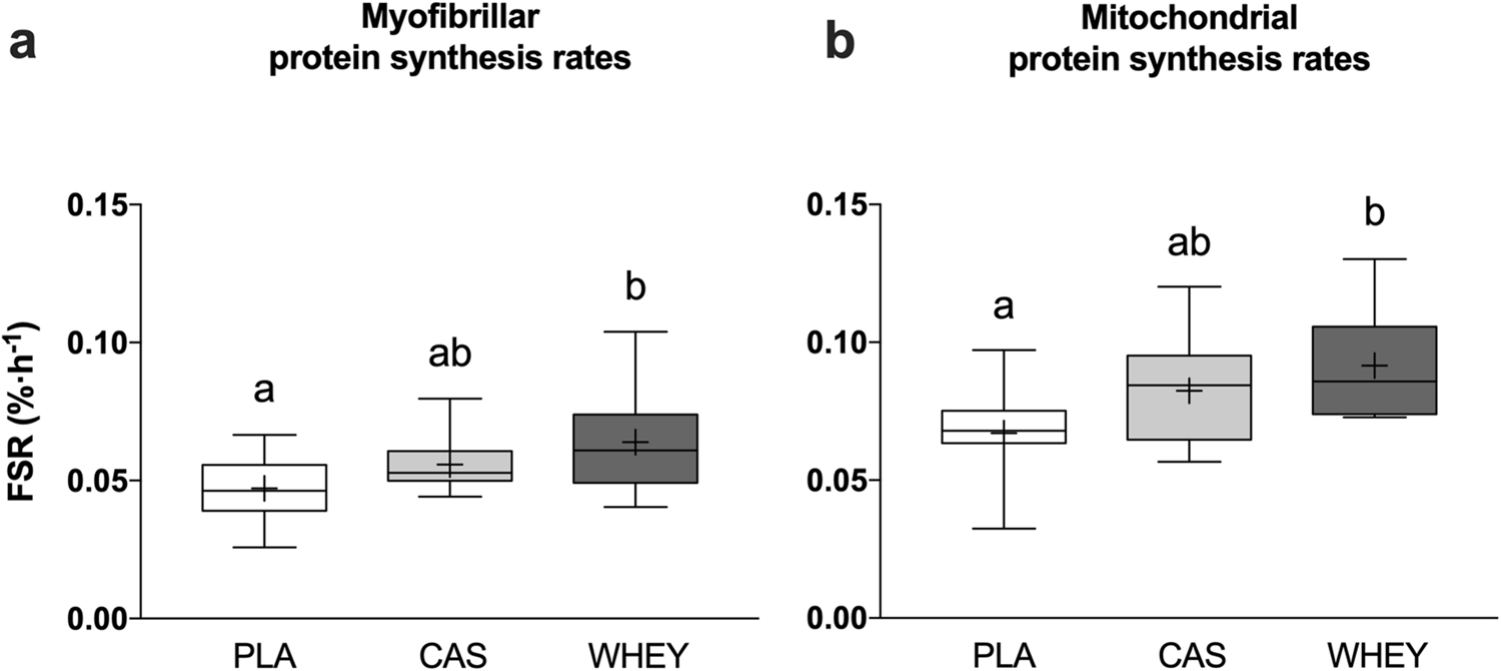
Overnight myofibrillar (**a**) and mitochondrial (**b**) protein synthesis rates following pre-sleep placebo (PLA), casein protein (CAS), or whey protein (WHEY) ingestion. The data are presented as box-and-whisker plots with the median (line), mean (cross), interquartile range (box), and minimum and maximum values (tails). Data were analyzed with a one-way analysis of variance with treatment as the between-subject factor and a Bonferroni correction was applied. Analysis of variance: panel **a**: *p* = 0.015, panel **b**: *p* = 0.009. Treatments without a common letter differ, *p* < 0.05. *FSR* fractional synthetic rate

Overnight myofibrillar protein synthesis rates (Fig. 5a) were significantly different between treatments (0.047 ± 0.011, 0.056 ± 0.009, and 0.064 ± 0.018%·h^−1^ in PLA, CAS, and WHEY, respectively, ANOVA: *p* = 0.015). We collapsed the data from CAS and WHEY into a single treatment group (PROTEIN) to compare the impact of protein ingestion versus placebo ingestion. Overnight myofibrillar protein synthesis rates were significantly higher in the PROTEIN treatments (0.060 ± 0.014%·h^−1^) when compared with the PLA treatment (*p* = 0.012). When comparing individual treatments, overnight myofibrillar protein synthesis rates were significantly higher in the WHEY treatment when compared with PLA treatment (*p* = 0.012). CAS ingestion resulted in intermediate values that were not significantly different when compared to either the PLA or WHEY treatment.

Overnight mitochondrial protein synthesis rates (Fig. 5b) were significantly different between treatments (0.067 ± 0.016, 0.082 ± 0.019, and 0.092 ± 0.020%·h^−1^ in PLA, CAS, and WHEY, respectively, ANOVA: *p* = 0.009). Overnight mitochondrial protein synthesis rates were significantly higher in the PROTEIN treatments (0.087 ± 0.020%·h^−1^) when compared with the PLA treatment (*p* = 0.005). When comparing individual treatments, overnight mitochondrial protein synthesis rates were significantly higher in the WHEY treatment when compared with PLA (*p* = 0.008). CAS ingestion resulted in intermediate values that were not significantly different when compared to either the PLA or WHEY treatment.

### Muscle Fraction Purity

**Fig. 6:**
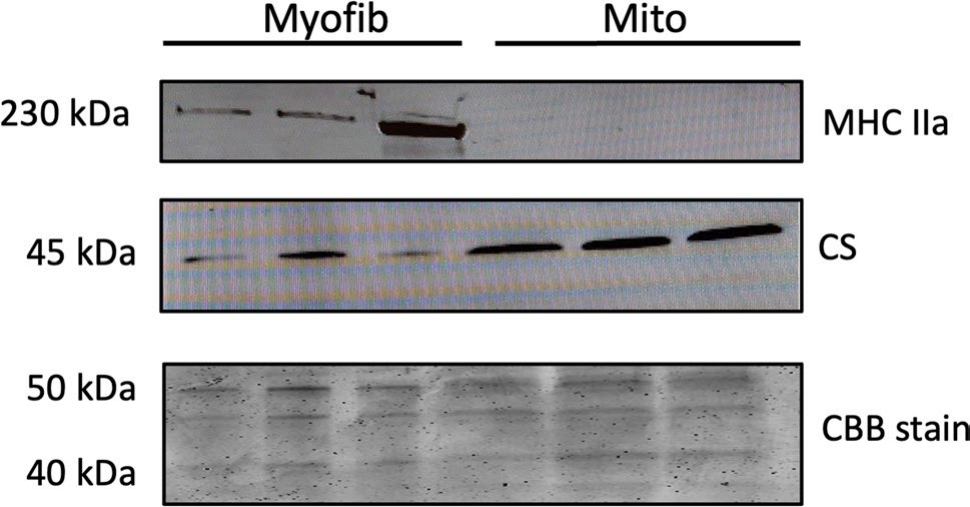
Myosin IIa (MHC IIa) and citrate synthase (CS) protein abundance in myofibrillar (Myofib) and mitochondrial (Mito) protein fractions (**a**), equal amount of protein loaded on the gel was confirmed by staining the SDS-page gel (**b**). *CBB* coomassie brilliant blue

MHC IIa protein was identified in the myofibrillar protein fraction, but absent in the mitochondrial protein fraction (Fig. 6). Citrate synthase protein was slightly present in the myofibrillar protein fraction, but was substantially higher in the mitochondrial protein fraction. In line, maximal citrate synthase activity was much higher in the mitochondrial protein fraction when compared with the myofibrillar protein fraction (2727 ± 1047 and 63 ± 49 μmol·g^−1^·min^−1^).

### Sleep, Hunger, and Energy Intake

The feeling of sleep (visual analog scale sleep score) was decreased in the morning when compared with prior to sleep (time effect: *p* < 0.001), with no differences in this increase between treatments (time × treatment interaction: *p* = 0.085). The visual analog scale hunger scores did not change over time or between treatments (*p* > 0.05). Energy intake averaged 31 kJ·kg body mass^−1^ during the ad libitum breakfast with no differences between treatments (*p* = 0.561).

## Discussion

The present study showed that pre-sleep protein ingestion stimulates myofibrillar and mitochondrial protein synthesis during overnight recovery from endurance-type exercise. Furthermore, we demonstrate that casein protein ingestion prior to sleep does not provide a greater anabolic response during overnight sleep when compared to the ingestion of whey protein.

It has been well established that micellar casein is a slowly digestible protein source and that its ingestion results in a more sustained release of protein-derived amino acids in the circulation [8, 9]. In agreement with our previous work [1–3], we observed that the ingestion of micellar casein protein prior to sleep resulted in sustained hyperaminoacidemia throughout the entire overnight period (Fig. 2). So far, studies examining the plasma amino acid and anabolic response to pre-sleep protein ingestion have been limited to micellar casein protein. Whey is a fast digestible protein and its ingestion will result in a more rapid but transient increase in plasma amino acid levels when compared with the ingestion of casein protein [8, 9]. Indeed, whey protein ingestion prior to sleep resulted in a greater initial rise in circulating plasma amino levels and lower levels upon waking when compared with micellar casein protein (Fig. 2). Despite this, whey protein ingestion resulted in significantly higher plasma amino acid levels during most of the overnight period when compared with placebo ingestion (5.5 out of 7.5 h). Our data show that both rapidly and more slowly digestible proteins can be applied effectively to elevate plasma amino acid availability throughout overnight sleep.

To study the impact of the overnight increase in plasma amino acid availability on overnight muscle protein synthesis rates, we applied l-[^13^C_6_]-phenylalanine infusions and collected muscle biopsies before and after sleep (Fig. 1). We have previously shown that protein ingestion prior to sleep increases overnight muscle protein synthesis rates at rest and during recovery from resistance-type exercise [1, 3]. In the present study, we confirm previous findings and extend on these findings by showing that protein ingestion prior to sleep also increases myofibrillar protein synthesis rates during overnight recovery from endurance-type as opposed to resistance-type exercise (Fig. 5a). As the muscle protein synthetic response to protein ingestion can be modulated by the type of ingested protein, we compared the potential impact of whey versus casein protein ingestion. Overnight myofibrillar protein synthesis rates were ~ 18 and 35% higher following casein and whey protein ingestion when compared with placebo (Fig. 5a). There were no differences in the anabolic response to pre-sleep whey versus casein protein ingestion. These data are in line with previous observations of which two studies demonstrated greater postprandial muscle protein synthesis rates following the ingestion of whey protein when compared with casein [14, 18], but most studies have failed to observe significant differences [12, 13, 15–17]. As myofibrillar protein synthesis rates were numerically higher following whey protein ingested when compared with casein protein, our data strongly challenge the common belief that casein is the preferred protein source to stimulate overnight anabolism.

In addition to the impact of pre-sleep protein ingestion on overnight myofibrillar protein synthesis rates, we also assessed the impact on mitochondrial protein synthesis rates. Previous work has failed to detect an impact of post-exercise protein ingestion on mitochondrial protein synthesis rates [12, 20–24, 39]. All these studies have assessed the impact of protein ingestion on mitochondrial protein synthesis rates in the first few hours (4–6 h) following exercise. However, it appears that the protein synthetic response to exercise is more delayed for mitochondrial protein when compared with myofibrillar protein [21, 25–27]. The overnight recovery period may cater for the proposed latency in the exercise-induced stimulation of mitochondrial protein synthesis rates. We provided the protein supplements prior to sleep and assessed mitochondrial protein synthesis rates over a 7.5-h overnight period (2.5–10 h post-exercise). As a consequence, we demonstrate that pre-sleep protein ingestion stimulates overnight mitochondrial protein synthesis rates during overnight recovery. Overnight mitochondrial protein synthesis rates were 23% and 37% higher following casein and whey protein ingestion when compared with placebo (Fig. 5b). No significant differences in overnight mitochondrial protein synthesis rates were observed following whey versus casein protein ingestion. Our data provide the first evidence that post-exercise mitochondrial protein synthesis rates can be modulated by protein ingestion.

Our observations suggest that pre-sleep protein ingestion represents an effective strategy to support muscle tissue repair and facilitate the skeletal muscle adaptive response to endurance training. The observed increase in both myofibrillar as well as mitochondrial protein synthesis rates provides the rationale for the observation that protein supplementation can improve gains in aerobic capacity and lean mass during endurance training [40–42]. However, it should be noted that other studies have failed to observe benefits of protein supplementation on aerobic capacity following a period of prolonged endurance exercise training [43–45]. Nonetheless, from our findings, it is clear that pre-sleep protein ingestion may facilitate skeletal muscle conditioning following endurance training and may help athletes to improve endurance training efficiency. As endurance athletes typically do not consume much protein prior to sleep [46], pre-sleep feeding represents an important and often overlooked opportunity to consume a protein-rich meal or snack.

## Conclusions

This is the first study to show that pre-sleep protein consumption increases both mitochondrial and myofibrillar protein synthesis rates during overnight recovery from endurance exercise. Furthermore, we conclude that pre-sleep casein protein ingestion is not preferred over whey protein as a means to further increase post-exercise muscle protein synthesis rates during overnight sleep.

## Supplementary Information

Below is the link to the electronic supplementary material.

Supplementary file1 (PDF 216 KB)

## Declarations

### Availability of data and material

Available upon request.
