# Supplementary material for: Pre-sleep Protein Ingestion Increases Mitochondrial Protein Synthesis Rates During Overnight Recovery from Endurance Exercise: A Randomized Controlled Trial
Source: Sports Med. 2023 Mar 1;53(7):1445–55. doi: 10.1007/s40279-023-01822-3 (PMC10289916; doi:10.1007/s40279-023-01822-3)
Supplement: Supplementary file 1 — Supplementary file1 (PDF 216 KB) [file 40279_2023_1822_MOESM1_ESM.pdf]

Pre-sleep protein ingestion increases mitochondrial protein synthesis rates during overnight recovery from endurance exercise: a randomized controlled trial

Sports Medicine

Jorn Trommelen[1], Glenn A.A. van Lieshout[1,2], Pardeep Pabla[3], Jean Nyakayiru[2], Floris K. Hendriks[1], Joan M. Senden[1], Joy P.B. Goessens[1], Janneau M.X. van Kranenburg[1], Annemie P. Gijsen[1], Lex B. Verdijk[1], Lisette C.P.G.M. de Groot[4], and Luc J.C. van Loon[1,*]

[1] *Department of Human Biology, NUTRIM school of Nutrition and Translational Research in Metabolism, Maastricht University Medical Centre+, Maastricht, The Netherlands*

[2] *FrieslandCampina, 3818 LE, Amersfoort, the Netherlands*

[3] *MRC/Versus Arthritis Centre for Musculoskeletal Ageing Research, School of Life Sciences, University of Nottingham, United Kingdom*

[4] *Division of Human Nutrition, Wageningen University, Wageningen, the Netherlands.*

***Correspondence**: Luc J.C. van Loon, Ph.D.
Department of Human Biology
NUTRIM School of Nutrition and Translational Research in
Metabolism, Maastricht University Medical Centre
P.O. Box 616, 6200 MD Maastricht
The Netherlands
Tel: +31 43 3881397
Fax: +31 43 3670976
E-mail: L.vanLoon@maastrichtuniversity.nl

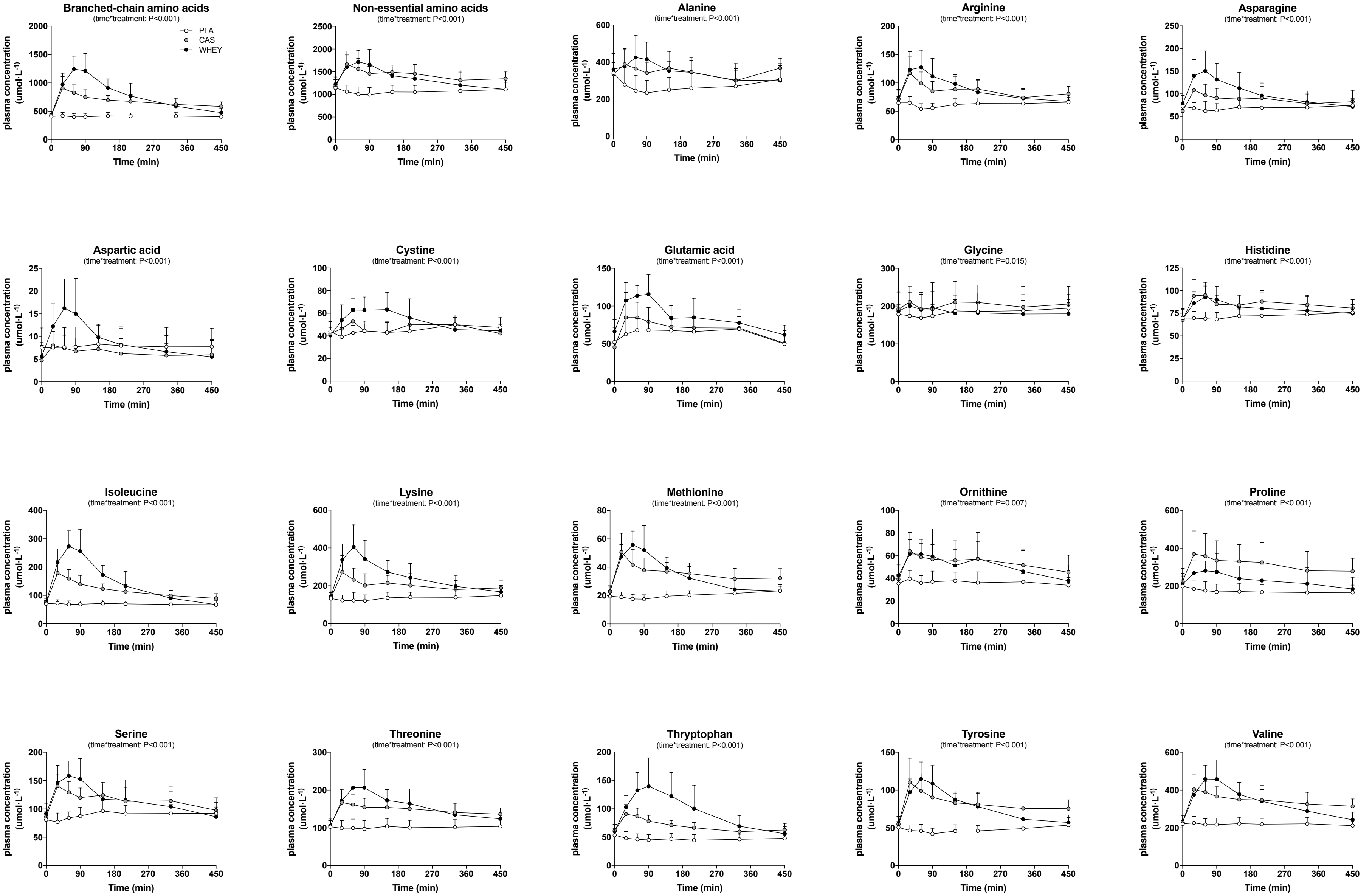
Branched-chain amino acids
(time*treatment: P<0.001)
Non-essential amino acids
(time*treatment: P<0.001)
Alanine
(time*treatment: P<0.001)
Arginine
(time*treatment: P<0.001)
Asparagine
(time*treatment: P<0.001)
Aspartic acid
(time*treatment: P<0.001)
Cystine
(time*treatment: P<0.001)
Glutamic acid
(time*treatment: P<0.001)
Glycine
(time*treatment: P=0.015)
Histidine
(time*treatment: P<0.001)
Isoleucine
(time*treatment: P<0.001)
Lysine
(time*treatment: P<0.001)
Methionine
(time*treatment: P<0.001)
Ornithine
(time*treatment: P=0.007)
Proline
(time*treatment: P<0.001)
Serine
(time*treatment: P<0.001)
Threonine
(time*treatment: P<0.001)
Thryptophan
(time*treatment: P<0.001)
Tyrosine
(time*treatment: P<0.001)
Valine
(time*treatment: P<0.001)
PLA
CAS
WHEY
plasma concentration (umol·L-1)
Time (min)
